# Stressful Experiences of Parents in the Paediatric Intensive Care Unit: Searching for the Most Intensive PICU Stressors

**DOI:** 10.3390/ijerph191811450

**Published:** 2022-09-12

**Authors:** Ivana Debelić, Anamaria Mikolčić, Jovana Tihomirović, Iva Barić, Đurđica Lendić, Željka Nikšić, Barbara Šencaj, Robert Lovrić

**Affiliations:** 1 School of Nursing, Medicinska Škola Osijek, 31000 Osijek, Croatia; 2 Nursing Institute “Professor Radivoje Radić”, Faculty of Dental Medicine and Health Osijek, Josip Juraj Strossmayer University of Osijek, 31000 Osijek, Croatia; 3 Specialist Practice of Occupational and Sports Medicine, Ilija Celebic, 31000 Osijek, Croatia; 4 Paediatric Clinic, University Hospital Centre Osijek, 31000 Osijek, Croatia; 5 Department of School Medicine, Teaching Institute of Public Health for the Osijek-Baranya County, 31000 Osijek, Croatia

**Keywords:** paediatric intensive care units, hospitalized child, parents, psychological stress

## Abstract

Hospitalization of a child in the paediatric intensive care unit (PICU) is extremely stressful, both for the child and for his or her family. The purpose of this study was to gain deeper insight into the stressful experiences of parents of children hospitalized in the PICU. This study included 96 parents. The data were collected using a translated and standardized scale “The Parental Stressor Scale: Paediatric Intensive Care Unit (PSS: PICU)”. This study confirms high exposure of parents to numerous PICU stressors. The most intense PICU stressor for parents was child’s breathing depending on the ventilator (4.22 ± 1.17), and the least intense was child’s demanding behaviour (1.17 ± 0.33). A significant positive correlation between the level of parents’ perceived stress and the number of their children was recorded (r = 0.240, *p* = 0.02), while there was no significant correlation between the level of stress and other sociodemographic variables. A significantly higher level of stress was experienced by parents with primary school education (*p* = 0.032) and parents who are not healthcare professionals (*p* < 0.01). It is necessary to establish a system that will enable continuous assessment of parents’ stress levels and timely prevention of stressful experiences for parents in the PICU.

## 1. Introduction

Rapid development of medical science and technology has caused numerous changes in the field of paediatric intensive care, which are directly related to positive outcomes regarding life and death of a hospitalized child [1]. However, one thing remains the same, and that is the unique experience of paediatric intensive care unit (PICU) hospitalization for children and their parents/caregivers (hereinafter referred to as Parents) [1]. Hospitalization of a child in the PICU is an extremely challenging, stressful, and even traumatic occurrence, both for the child and for his or her family [1,2]. An illness or injury may lead to potentially life-threatening condition in a child [3], which results in various uncomfortable feelings in parents caused by uncertainty and severe stress [2]. Hospitalization of a child in the PICU also significantly affects mental health of parents, their family structure and functioning, which additionally causes numerous negative short-term and long-term consequences (e.g., various forms of psychological illnesses, social contact disorders, carrying vivid memories, job loss, etc.) [2,4,5]. Despite the growing population of children hospitalized in the PICU and previous studies, there is insufficient information and knowledge about parents’ experiences of this traumatic event that can lead to long-term trauma and stress [6]. A significant number of studies have been conducted in neonatal intensive care units (NICU) [7,8,9,10], while notably fewer studies have included parents in the PICU [6,11,12], which is the primary focus of this study. 

The beginnings of research on parents’ stress during their child’s stay in the PICU date back to the 1980s [11,13]. Even then, the feeling of helplessness and changes in the child-parent relationship were singled out as more significant sources of stress in comparison to the physical environment in the PICU [13]. For parents, the PICU is a strange and illusory place, and the admission of a child to such an environment most often represents a new, unknown, somewhat unreal, chaotic, and extremely stressful situation [12]. Bright lights, fast-paced work and movement, loud noises, and interventions aimed at saving children’s lives have a significant impact on parents, for whom the very fact that their child is in danger of death is a stressful situation [14]. In such circumstances, various emotional, physical, and psychological problems can be identified in parents as early as 24 h after admission and may last for years after the child’s discharge [1]. They experience a hurricane of emotions [6]: shock, guilt, loneliness, fear, sadness, and anger [15,16], as well as symptoms of anxiety and depression [2,16,17]. In their study, Stremler et al. [2] describe how 24% of parents felt anxiety, about 51% had symptoms of depression, and 26% had significant difficulties in making decisions. Rodríguez-Rey et al. [3] suggest that parents experience equal, moderate to severe levels of anxiety, depression, and post-traumatic stress disorder three and six months after their child’s discharge from the PICU. The results of a retrospective study conducted in the USA on a sample of 95,070 parents indicate that mothers, compared to fathers, had mental health problems twice as often, six months after the child’s discharge from the PICU [18]. According to the same study, the need for prescribed medications for mood disorders in parents grows proportionally to the child’s length of stay (LOS) in the PICU [18]. Comparing data regarding general population and the population of children hospitalized in the PICU and their parents, higher rates of acute and post-traumatic stress are recorded in all children and their parents in the latter group [19]. The stress level of parents in the PICU is additionally intensified by the perception of the child’s grave illness, their constant concern about possible complications of the disease [20], and frequent invasive medical procedures [21]. Also, it is recorded that the stress level of parents increases with the frequency and duration of the child’s hospitalization [20]. In their respective studies, Hagstrom [22] and Alzawad et al. [6] describe parents’ experiences in the PICU as ‘riding a roller coaster’ due to experiencing unpredictable, helpless, better and worse moments, after which parents reported that the cause of the most intense stress for them was the condition of the child, not the PICU environment [6]. However, confusing and frightening aspects, such as noise and privacy restrictions, still contribute to stress levels, even if parents expect them [6]. Very similarly, Hagstrom detects three main causes of parental stress, among which are the child’s illness and the uncertainty of the treatment and recovery process [22]. At the beginning of the child’s hospitalization in the PICU stress is usually associated with the uncertainty of the child’s condition, treatment, and possible complications, and later it is more significantly associated with concern about the possible transition of the child’s acute illness to chronic one and its long-term outcomes [15]. Also, additional unnecessary stress for parents in the PICU can be caused by healthcare professionals who diminish the role of parents in providing care for the child [15]. Psychological outcomes of the parents became worse with the child’s health condition being more difficult [2]. Moreover, a positive correlation between the psychological outcomes of parents and their children has been noticed [17]. According to Yagiela et al. [17], negative psychological outcomes correlate with extreme stress, previously experienced stressors and parental trauma, and stress reactions. Families of children placed in the PICU often come from different cultural backgrounds, which can affect their reaction and ways of coping with a sudden event and newly developed stressful situation. This factor shows significant differences in developed countries in comparison to developing countries [23,24]. Also, parents’ previous traumas can influence their interpretation of stressors in the PICU [17]. The initial shock at the admission of the child and the subsequent feeling of helplessness leads to a state of psychological exhaustion for the parents, and the experiences of the parents are still traumatizing regardless of the ever-improving health care in the PICU [6]. 

It is evident that the previously described knowledge and studies consider the stress of parents in the PICU from different aspects and indicate different and conflicting results, depending on the cultural background and specific family factors. Due to today’s intense social changes and the accelerated development of medical science and technology, it has become necessary to apply frequent and continuous strategies to assess the stress level of parents in the PICU as well as to detect certain specific PICU stressors, which can ultimately contribute to more effective prevention and alleviation of parental stress in the PICU. 

Therefore, the purpose of this study was to gain a deeper insight into the stressful experiences of parents of children hospitalized in the PICU. For this purpose, it was attempted: (a) to examine the exposure of parents to specific stressors in the PICU, (b) to examine the level of perceived stress of parents for certain PICU stressors, (c) to examine whether there is a correlation between the socio-demographic characteristics of parents and the level of experienced stress in the PICU, and finally, (d) to examine whether there are differences in the level of experienced stress with regard to the parents’ earlier experiences with stays in the PICU.

## 2. Materials and Methods

### 2.1. Theoretical Framework

The conceptual framework for this study is based on Lazarus and Folkman’s Transactional Theory of Stress and Coping [25]. According to this theory, the person and the environment are in a dynamic, two-way relationship, and psychological stress is defined as a burdensome relationship between the environment and the person, which threatens personal well-being and exceeds one’s own resources [25]. Studying stress from a psychological perspective, Lazarus singles out harm, challenge, and threat as three types of stress [26]. He, along with Folkman, observes that the direct psychological consequences are caused by our thoughts and understandings of situations [25]. In fact, psychological stress is the result of different threats and harms resulting from the relationship between the environment and the person, while the reaction to stress is conditioned by assessment and coping, which are also influenced by personal characteristics and environment [26]. The cognitive assessment determines a person’s understanding of the situation and of what a person can do about it. A perception and anticipation of possible harm and threat can significantly impair one’s own mental functions [25,26]. Transactional Theory of Stress and Coping is often the theoretical starting point for the studies of stress in children and their parents/caregivers [6,27,28,29]. The theory interprets the psychologically stressful experience of parents in the PICU as a result of a complex transaction between the personal characteristics of the parents (e.g., age, gender, level of education, number of children in the family, etc.), certain situations that parents experience in the PICU (e.g., admission of the child to the PICU, severity of medical diagnoses and prognoses, etc.) and various environmental factors (e.g., monitor sounds, alarms, medical devices, etc.) [6,13].

### 2.2. Study Design

This cross-sectional study was conducted at the Clinical Hospital Centre Osijek (CHC), Croatia. Criteria for choosing the mentioned health institution for our study were: CHC in our study is the central and largest health institution in this region of our country. The PICU in the examined health institution treats all life-threatening injuries and/or sick children covering a large area of the eastern part of Croatia and neighbouring countries. CHC is also the largest health care institution and the main teaching base for health professionals in this region.

### 2.3. Respondents

Respondents were 96 parents or guardians of children hospitalized in the PICU. Criteria for the parents’ inclusion in the research were: their child was under 18 years of age, the child’s length of stay in the researched PICU was for a minimum of 48 h, and their participation in the research was voluntary. The exclusion criteria of the respondents were: insufficient knowledge and use of Croatian language to fill in the questionnaire, previous existence of acute psychiatric symptoms, refusal to participate in the research, immediate death of a child, and suspicion of child abuse.

### 2.4. Instrument

The data were collected using a translated and standardized scale *The Parental Stressor Scale: Pediatric Intensive Care Unit* (PSS: PICU) by Margaret S. Miles, School of Nursing, University of Kansas, who gave written permission for translation and use of the scale [29]. PSS: PICU is currently the only validated instrument available to assess parent-reported PICU stressors [30]. Also, the Croatian version of the PSS: PICU instrument, with its content, scope, and psychometric properties, fully meets the set goals of this study. The Cronbach’s alpha of the subscales ranges from 0.72 to 0.99, while the alpha coefficient of the entire scale is 0.95 [29].

Thirty-nine items of the questionnaire represent specific factors for which parents of children hospitalized in the PICU experience a certain level of stress (PICU stressors). PICU stressors are organized into seven subscales: ‘Child’s appearance’ (1–3), ‘Sights and sounds’ (4–7), ‘Procedures in PICU’ (8–14), ‘Staff communication’ (15–19), ‘Child’s behaviour and emotional responses’ (20–29), ‘Behaviours of professional staff’ (30–33), ‘Parental role alteration’ (34–39).

In the first phase, the forward translation of the questionnaire from English to Croatian was done by three independent translators, with the help of an English teacher (native Croatian speaker). None of the translators had seen the PSS: PICU instrument before. In the second phase, the back-translation was conducted by an independent translator (a bilingual native English speaker living in Croatia). Finally, all the translators and authors of the article analysed and evaluated the original and back-translation of the questionnaire and agreed on the final version [31,32]. 

The assessment of the content validity of the Croatian version of PSS: PICU was done as recommended by Reichenheim et al. and Beaton et al. [33,34]. Formed an expert committee (four translators, one methodologist, one assistant professor in the Croatian language, having a proficient knowledge of English, four assistant professors in the field of nursing, and one psychologist) independently assessed whether the synthesized translated version adequately represents the same meaning and relevance, in addition to the acceptability from a linguistic perspective [31,35].

Finally, several randomly selected volunteers reviewed the instrument, checking each item for precision and clarity of the questions asked. The volunteers were parents who were selected as the respondents but did not participate in this study [31,32,35]. After the selection process, parents assessed the comprehensibility of the introductory instructions and the complete content (all the items) in the instrument. Upon completion of the assessment, parent volunteers provided the researchers with feedback on the content of the questionnaire. 

The reliability of the Croatian version of the PSS: PICU scale was tested using Cronbach’s alpha coefficient. Each subscale shows a high level of reliability: 0.802 (‘Child’s appearance’), 0.871 (‘Sights and sounds’), 0.862 (‘Procedures in PICU’), 0.892 (‘Staff communication’), 0.868 (‘Child’s behaviour and emotional responses’), 0.827 (‘Behaviours of professional staff’), 0.900 (‘Parental role alteration’). Overall reliability of the Croatian version PSS: PICU is 0.948, which indicates the high reliability of this instrument. The parent’s answers to the questionnaire (level of experienced stress for a particular stressor) were evaluated on a five-degree Likert scale. Each item is scored from 1 to 5 points (not at all stressful =1, a little stressful = 2, moderately stressful = 3, very stressful = 4, extremely stressful = 5). For all 39 PICU stressors, respondents additionally had the option to circle N/A (Not Applicable) if they did not have any previous experience with a particular stressor. The introductory part of the instrument included questions for collecting demographic and other general data (e.g., gender, age, level of education, place of residence, marital status, number of children, earlier stays in PICU, and self-assessed social status).

### 2.5. Data Collection

The data were collected during a six-month period in the PICU as part of the paediatric clinic in the aforementioned health facility. The respondents voluntarily participated in the research. Researchers (authors of this manuscript) distributed the questionnaires to respondents who voluntarily filled in the questionnaires using the pen and paper method. According to defined research criteria, parents filled in the questionnaires on the third day of their stay at the PICU. Parents filled in the questionnaire in the room of their hospitalized child at the time intended for daily rest. In the case of certain personal or situational factors that prevented the parents from filling in the questionnaire in the allotted time, the parents filled in the questionnaire no later than the fifth day of their stay in PICU. The time for completing the questionnaire was not limited; it lasted 30 min on average. 

During the research period, a total of 142 children were hospitalized in the PICU. Of those 142, 8 stayed in the PICU for less than 48 hours, which was the exclusion criterion. The remaining 134 parents were offered participation in this study, and 102 of them were interested in completing the questionnaires, which accounted for a 76.1% response rate. All parents understood and spoke Croatian and had no difficulties while filling in the questionnaire. Subsequently, 6 anonymous questionnaires were excluded from further analysis because they were not completely and/or correctly filled in, which makes the final sample of 96 analysed questionnaires/parent respondents.

### 2.6. Data Analysis

Descriptive statistics for nominal variables were performed and data are presented as shares and percentages, and numerical data with basic measures of average value and dispersion (arithmetic mean and standard deviation). The Shapiro-Wilk test was used to test the normality of the distribution of numerical variables. Unpaired Student’s t-test was used to examine differences between two independent groups. Therefore, the parametric One-way analysis of variance test (ANOVA) was used to compare the mean differences among several independent groups. Pearson’s correlation coefficient (r) was used to calculate the association between two variables in a linear relation. The statistical reliability of the instruments was tested using Cronbach’s alpha coefficient. The significance level α = 0.05 was used as criteria for the statistical significance of the obtained results. SPSS for Windows software (version 22.0, IBM SPSS, Armonk, NY, USA) was used for data analysis. 

### 2.7. Ethical Considerations

Before each data collection, the researchers informed the respondents in detail about the purpose and research objectives, all ethical issues, and details of the PSS: PICU scale. The respondents had the right to withdraw from the study before and during filling in the questionnaire. Respondents were guaranteed anonymity, i.e., it was made impossible to establish their identity from the answers. Only researchers had access to the research data. The researchers got the consent of the author of the original version of the PSS: PICU scale for the translation and use of the PSS: PICU as an instrument in this study. The study was approved by the Ethical committee of the institution where the study was conducted (IRB approval number: R1-10473-4/2019).

## 3. Results

### 3.1. Sociodemographic Characteristics of Respondents 

Out of the total of 96 respondents, 60 (62.5%) were female, and 36 (37.5%) were male parents (Table 1). The average age was 34.27 ± 6.59 years with a range of 22–50 years. Most respondents, 54 (56.3%), lived in rural areas. They were mostly high-school graduates, 60 (62.4%). Most of them, 91 (94.8%), were not healthcare professionals. Most of them, 72 (75%), were married and the majority of respondents, 39 (40.6%) had two children. This was the first experience of staying in the PICU for 62 (64%) respondents. Parents in the largest number, 57 (59.4%) self-assessed their social status as satisfactory.

### 3.2. Exposure and Level of Perceived Stress of Parents of Children Hospitalized in the PICU

The overall mean of the level of perceived stress of parents according to the PSS: PICU (response range 1–5) was 2.40 ± 1.32. Furthermore, the analysis of specific subscales of PSS: PICU scale indicated significant differences (*p* < 0.01) (Table 2).

A significantly highest level of parents’ experienced stress was expressed for the subscale ‘Parental role alteration’ (3.00 ± 1.30), while the lowest level of stress was expressed for the subscale ‘Behaviours of professional staff’ (1.75 ± 1.00) (Table 2).

Detailed analysis of mean values of parents’ perceived stress for individual PICU stressors showed that among the first 10 most stressful factors there were five from the ‘Parental role alteration’ subscale: *Not knowing how best to help my child during this crisis* (3.24 ± 1.21), *Not being able to see my child when I wanted* (3.14 ± 1.25), *Not being able to hold my child* (3.10 ± 1.26), *Not being able to visit my child when I wanted* (3.09 ± 1.27), and *Not being able to be with my crying child* (2.86 ± 1.40) (Table 3). Also, among the 10 most stressful stressors for parents there were three from the ‘Procedures in PICU’ subscale: *Having a machine (respirator) breathe for my child* (4.22 ± 1.17), *Putting needles in my child for fluids, procedures, or tests* (3.08 ± 1.36) and *Tubes in my child* (2.90 ± 1.42), and then two PICU stressors from two subscales: *The sudden sounds of monitor alarms* (3.24 ± 1.43) (subscale ‘Sights and sounds’) and *Acting or looking as if in pain* (3.18 ± 1.27) (subscale ‘Child’s behaviour and emotional responses’). The intensity rank of experienced stress showed that the five most stressful PICU stressors for parents were *Having a machine (respirator) breathe for my child* (rank 1), *Not knowing how best to help my child during this crisis* (rank 2), *The sudden sounds of monitor alarms* (rank 3), *Acting or looking as if in pain* (rank 4) and *Not being able to see my child when I wanted* (rank 5).

Among the 10 least stressful PICU stressors for parents were four from the ‘Child’s behaviour and emotional responses’ subscale: *Demanding* (1.17 ± 0.33), *Rebellious or uncooperative behaviour* (1.21 ± 0.40), *Restlessness* (1.68 ± 0.85) and *Anger* (1.75 ± 0.81) (Table 3). Also, among the 10 of the least stressful stressors were three from the ‘Staff communication’ subscale: *Explaining things too fast* (1.65 ± 0.80), Not talking to me enough (1.77 ± 0.87), *Not telling me what is definitely wrong with my child* (1.79 ± 0.99; rank 24), and three PICU stressors from the subscale ‘Behaviours of professional staff’: *Not telling me their names or who they are* (1.54 ± 0.91), *Joking, laughing, or talking loudly* (1.75 ± 0.80) and *Insufficient communication with me* (1.79 ± 099). Intensity rank of stressful experiences indicated that the five least stressful PICU stressors for parents were: *Demanding* (rank 39), *Rebellious or uncooperative behaviour* (rank 38), *Not telling me their names or who they are* (rank 37), *Explaining things too fast* (rank 36), *Restlessness* (rank 35).

Analysis of the number (%) of parents who were exposed to certain PICU stressors indicated that all respondents (100%) experienced the three stressors: *Putting needles in my child for fluids, procedures, or tests*, *The other sick children in the room* and *Crying or whining* (Table 3). Furthermore, more than 90% of respondents were exposed to as many as 11 PICU stressors: Seeing the heartbeat on the monitors (98.9%), *The sudden sounds of monitor alarms* (98.9%), *Not knowing how best to help my child during this crisis* (96.9%), *Injections/shots* (96.8%), *Tubes in my child* (96.8%), *The sound of monitors and equipment* (96.8%), *Bruises, cuts, incisions on my child* (93.8%), *Not being able to visit my child when I wanted* (91.7%), *Not being able to see my child when I wanted* (91.7%), *Not being able to hold my child* (91.7%) and *Colour changes in my child (pale, blue, or yellow)* (91.6%) (Table 3). Less than 50% of respondents experienced only two PICU stressors: *Not telling me their names or who they are* (47.9%) and *Having a machine (respirator) breathe for my child* (18.8%), while more than 50% of respondents were exposed to the remaining 23 PICU stressors (Table 3).

### 3.3. The Relationship of Sociodemographic Characteristics and Situational Factors with the Experienced Stress of Parents in the PICU

A significant positive correlation between the level of parents’ perceived stress and the number of their children was recorded (r = 0.240, *p* = 0.02), while there was no significant correlation between the level of stress and other sociodemographic variables (age, gender, place of residence, social status, marital status) (Table 4).

Considering parents’ level of education, a significantly higher level of experienced stress was recorded (*p* = 0.032) in parents with completed primary school (2.67 ± 0.61) compared to parents with completed high school (2.04 ± 0.90) and parents having a university degree (1.70 ± 0.80) (Table 5). This was particularly evident in the subscales: ‘Child’s appearance’ (*p* < 0.01), ‘Sights and sounds’ (*p* < 0.01) and ‘Procedures in PICU’ (*p* = 0.023), where parents with completed primary school experienced significantly higher level of stress in comparison to more highly educated parents.

Furthermore, a significantly higher level of experienced stress was recorded (*p* < 0.01) in parents who are not healthcare professionals (2.03 ± 0.87) in relation to the stress level of parents who are healthcare professionals (0.93 ± 0.40) (Table 5). According to the subscales of PSS: PICU, parents who are not healthcare professionals expressed a significantly higher level of experienced stress for the subscales: ‘Procedures in PICU’ (*p* = 0.013), ‘Staff communication’ (*p* = 0.004), ‘Child’s behaviour and emotional responses’ (*p* = 0.024) and ‘Behaviours of professional staff’ (*p* = 0.014) (Table 5).

Considering the parents’ earlier experiences with stays in the PICU, the level of experienced stress was significantly higher (*p* = 0.022) in parents whose child was previously hospitalized in the PICU and this was their repeated stay (2.25 ± 0.93) in relation to the stress level of parents whose child was in the PICU for the first time (1.82 ± 0.82) (Table 5). Also, parents who had repeated stays in the PICU expressed a significantly higher level of stress for four subscales: ‘Child’s appearance’ (*p* = 0.013), ‘Procedures in PICU’ (*p* = 0.011), ‘Child’s behaviour and emotional responses’ (*p* = 0.017) and ‘Parental role alteration’ (*p* = 0.028).

## 4. Discussion

The purpose of this study was to get a deeper insight into the specifics of the stressful experiences of parents whose children were hospitalized in the PICU. The mean value of experienced parental stress measured using the PSS: PICU questionnaire was 2.40 (response range 0–5), which is in accordance with the results of related studies [36,37,38].

At first glance, the stated total mean value may seem low, however, it should be noted that the PSS: PICU questionnaire contains a large number of items (PICU stressors) that parents rated as very low stress, which ultimately significantly contributes to the overall mean values of experienced stress. However, a detailed analysis of the results at the PSS: PICU subscale and individual items/stressors level clearly indicate frequent exposure and very high levels of parents’ experienced stress for individual PICU stressors, which is described further below in the discussion section.

From the PSS: PICU subscale aspect, the most stressful one for parents was the subscale ‘Parental role alteration’, which is in line with a study conducted in India [21]. Similar studies also highlight this subscale as extremely stressful for parents [14,24], while parents in some studies have not found changes in their parental role more stressful than other PSS: PICU subscales [37,39]. According to the updated systematic review by Abela et al. [1], the impact of PICU stressors from this subscale begins the moment the child is hospitalized, and it leaves its consequences even after discharge when parents have to adapt to the new needs and problems of the child who suffered hospitalization in the PICU. The role of parents in the PICU is additionally influenced by environmental factors, such as numerous tubes and cables that prevent parents from freely moving, approaching, and helping their child [1]. Inability and not knowing how to help their own child, which is something that parents usually do every day under normal conditions, proved to be the most significant source of stress for parents in this study.

Furthermore, parents indicate that not being able to visit, see and hold their own child are the most stressful PICU stressors from this subscale, as well as not being present in moments when the child cries, which is in accordance with the results of a study conducted in India [37]. These results clearly indicate that PICU work structure needs to be organized in a way that allows parents to spend as much time as possible with their sick child as well as actively participate in the care of the child, so that their parental role deviates as little as possible from the usual one [14]. Many studies confirm the importance of parents being present and participating in health care of their child [40,41,42]. Also, many benefits of parental presence during invasive procedures in the PICU have also been reported for both the child and parents, including presence during resuscitation, regardless of the outcome [43]. Thus, it is easier for the child to stay in an unknown environment, because they have by their side a familiar face, someone whom they trust, and parents feel safer, knowing at all times what is happening with their child. Parental comfort, tenderness, and parent-child closeness are extremely important to the parents [40]. If children are separated from their parents, everyday social ties are disturbed, and naturally, children and parents can feel lonely and alienated [42].

The next most stressful PSS: PICU subscale for parents was ‘Procedures in PICU’, which is in compliance with the previous related studies in which parents also singled out this subscale as the most stressful [21,39]. Although in almost all studies available to us, a significantly smaller number of children required mechanical ventilation, this is the most intense PICU stressor for the parents [37]. Haines et al. [39] report different causes of stress in parents of intubated children, in contrast to parents of non-intubated children. Namely, parents of intubated children are more often negatively affected by painful medical procedures performed on their child, while the child’s behaviour and emotional response are the most stressful for parents of non-intubated children [39]. This study also indicated that for the parents of intubated children, a stay in the PICU was a stressful factor by itself, while parents of non-intubated children considered some of their environmental factors more important [39]. Furthermore, in this study, in the subscale ‘Procedures in PICU’ the item Putting needles in my child for fluids, procedures, or tests and the item Tubes in my child, were significantly stressful for parents, which is in line with other studies [21,39]. Abela et al. [1] report that witnessing certain medical procedures performed on children by medical personnel may have a significant influence on development of psychiatric disorders in parents and lead to post-traumatic stress disorder. In addition, the authors point out that numerous qualitative studies involving parents during their child’s hospitalization in the PICU, but also after their discharge, support these findings [1].

In the PICU, parents are put in a sudden situation, surrounded by unknown people and equipment that produces various sounds, which often has a disturbing effect on children and parents. It is the sudden sounds of the monitor that prove to be highly stressful for parents, which is in compliance with related studies [21,39]. In recent decades, there has been significant progress in the process of monitoring sick children, and it is necessary to explain PICU workflow to parents as well as the importance of certain devices and procedures, so that they cause minimal stress to the parents. A recent study conducted in India described the opposite results, according to which the mentioned PICU stressors had the least stressful impact on parents [21]. The researchers consider that such results were obtained due to effective communication and the fact that parents were informed and educated about certain diagnostic and therapeutic procedures, medical interventions, as well as details about specific equipment (e.g., airway, infusion system, needles, intravenous cannulas, nasogastric tubes, etc.), and medical devices (e.g., monitors, infusion pumps, respirators, suction pumps, etc.).

The least stressful PSS: PICU subscale according to the parents in this study was ‘Behaviour of professional staff’, which is in accordance with the results of a study conducted in Jordan, in which the parents specifically mentioned the behaviour and communication of the professional staff as the least stressful [24]. However, there are still a large number of studies in which parents perceive this subscale as significantly more stressful [14,21]. These results can be attributed to cultural differences related to social norms and professional behaviour of the staff towards patients and their relatives and the work organisation of individual PICUs [23]. In their study, Haines et al. [39] describe how the medical staff was more attentive to the parents of intubated children, compared to parents of non-intubated children, who were somehow “left to themselves and the monitor”. Parents, in such moments, feel neglected, and the medical staff leave the impression of being less caring [39]. Interestingly enough, according to Rodríguez-Rey et al. [14], the stress factors from this subscale have a relatively short-lived effect since their impact ends after the child is released from hospital.

Furthermore, parents from this study also found ‘Child’s behaviour and emotional responses’ as the least stressful PICU subscale, in which the least stressful items were Demanding, Rebellious or uncooperative behaviour, Restlessness, and Anger. The reason can be that this type of behaviour is relatively common, even expected in healthy children, especially younger ones. ‘Protest’ is one of the most expected child’s reactions to hospitalization and separation from parents [44]. Haines et al. [39] describe that ‘Behaviour of professional staff’, had a significant impact on the parents of non-intubated children, who paid more attention to the behaviour of the professional staff in the PICU. It is possible that these two subscales (‘Child’s behaviour and emotional responses’ and ‘Behaviour of professional staff’) correlate, since the staff behaviour can directly affect a child’s emotional response. Rodríguez-Rey et al. [14] also report that parents consider the child’s behaviour extremely stressful and that it is necessary to take action to prevent parents from witnessing the child’s suffering as much as possible. In this subscale, Acting or looking as if in pain was the biggest source of stress for the parents in our study and was generally among the first five most stressful according to the PSS: PICU scale. This can be attributed to the fact that parents often assessed the child’s condition and the severity of his or her illness or injury based on child’s appearance [21]. Changes in the child’s behaviour and appearance can be dramatic and stressful for the parents, especially when they notice that the child is in pain. In such moments they need to be with their child and actively participate in pain relief procedures [15]. Due to the disease itself and the need for invasive and painful procedures, the experience of pain in the PICU is inevitable and represents an important problem. In seriously ill children, the assessment and treatment of pain are a challenging part of health care, but their adequate implementation has a positive effect on the child and the parents [45].

The results of this study did not indicate ‘Staff communication’ as a significant source of stress for parents. In contrast, one study conducted in India [23] reported that professional communication of PICU staff caused acute stress in parents. The authors of the mentioned study believe that the presence of professional counsellors would contribute to solving this problem, as they could talk to parents, and help them work through their problems and worries [23]. Consistent with the results of the study by Pooni et al. [23], there is still chronic nursing shortage in Croatia today. According to the latest available data from OECD/EU, the Republic of Croatia has fewer nurses than the European average [46].

Hospital staff often includes psychologists and occupational therapists, professionals whose task is to alleviate the psychological stress of doctors and nurses. Nurses and doctors in the PICU are often stretched between numerous patients and obligations, but also parents, who would like to have the opportunity to share their worries and problems with someone. The results of other related studies considering the stressor of ‘Staff communication’ in the PICU are different and divided. Some studies single out the professional communication of staff as a significant stressor for parents in the PICU [14,39], while in other studies parents do not find this PICU subscale as stressful [4,21]. For parents, the least stressful item from this subscale is Explaining things too fast, which can be explained by the fast-paced work in the PICU. Healthcare workers are often expected to react extremely quickly in acute situations and they do not have enough time to explain in detail and potentially repeat certain information to parents [47]. A higher level of stress in this subscale can be prevented by better communication with parents in moments when the situation is calmer and there is not a life-threatening urgency. The staff should then try to find time to talk to the parents, answer their questions and resolve all doubts that they might have.

The results of this study showed a positive correlation between the level of parental stress and the number of their children, which is contrary to the results of another study in which parental stress did not correlate with the number of children [23]. Hospitalization of a child often leaves a mark on the parent’s relationship with their healthy children, because their time is mostly spent on providing support to the sick child [1]. Parents who stay with a sick child in the PICU spend less time at home with the rest of the family. This change in family dynamics can negatively affect their relationship with their partner and other children, who can potentially feel neglected [1,22]. Although this aspect is often overlooked due to the acute situation with a sick child, parents also must not forget to pay attention to their healthy children. Siblings are also active participants in the current situation, often witnessing the suffering of a sick child and their own parents.

The results of this study also indicated a negative correlation between the parent’s level of education and their level of experienced stress. Parents with primary school education expressed a significantly higher level of stress compared to more highly educated parents. These results deviate notably from the results of related studies in which highly educated parents experience a higher level of stress [36,48]. Ramírez et al. [48] explain their results by stating that parents with a higher level of education are expected to be more competent in processing and collecting information, and to better understand the child’s illness and treatment procedures, which in a way also explains the opposite results of this study. It can be assumed that parents with primary education do not understand the seriousness of the situation in which their child is and also the information that is provided to them about the disease and the treatment, which in turn leads to an increase in their stress level. Also, considering the fast-paced work in the PICU, parents may not have enough time, will, or courage to ask for additional clarification, while healthcare professionals may overlook their need for additional information for the same reason. In the aforementioned study conducted in India, highly educated parents also expressed dissatisfaction with the behaviour of healthcare staff more often, but the level of education did not correlate with the overall level of experienced stress [23]. Nelson et al. [19] also state that parents with university-level education developed a significantly higher degree of post-traumatic stress after discharge from hospital in contrast to parents with primary or secondary education.

Parents who are not healthcare professionals, and participated in this study experienced a significantly higher level of stress in relation to the parents who are medical professionals, which is not surprising because their expectations were probably completely different. It can be assumed that the parents who are medical professionals not only possess medical knowledge and skills, but also have experience from their workplace and have been more or less exposed to the stressors of the clinical environment (medical equipment and accessories, invasive medical technical procedures, diagnostic and therapeutic procedures). Therefore, their expectations were probably based on earlier professional experiences, which helped them to cope better with specific PICU stressors. Available related studies do not offer similar comparisons and explanations.

This study did not indicate a significant correlation between age, gender, place of residence, social and marital status with the level of parents’ experienced stress, which partially differs from the results of previous studies in which at least one of the mentioned variables proved to be significant [4,14,23]. Although fathers were often less represented in related studies, their stress level was often higher in certain PSS: PICU subscales [4,24]. Alzawad et al. [4] state that healthcare professionals should not assume that the level of stress would be higher in one of the parents based on their gender. When it comes to comparing mothers of children hospitalized in the PICU and those hospitalized in general paediatric care units, the mothers in the first group expressed a significantly higher level of anger, confusion, depression, and anxiety [37]. In the study conducted by Pooni et al. [23], significantly higher level of stress was recorded in mothers compared to fathers. The authors also state that studies conducted in developed countries have shown that although the level of stress in both parents is equal, the influence of individual stressors differs according to gender.

The results of this study indicated a higher level of stress in parents who experienced a repeated stay in the PICU, which differs from related studies in which there was no detected correlation between previous PICU hospitalizations and higher levels of stress in parents [14,37]. Ramírez et al. [48] used a very similar scale to assess parental stress in the PICU in their study, which indicated that a child’s multiple hospitalizations in the PICU is associated with higher levels of stress in parents. Also, they state that previous PICU hospitalizations are often considered a protective factor against parents’ stress [48]. It can be assumed that parents, due to previous bad experiences, both personal and related to other seriously ill children and their parents in the PICU, feel fear and concern, and consequently, they report a higher level of stress.

### 4.1. Limitations of the Study

The study has certain limitations. Firstly, the research was conducted in a single clinical hospital centre. It would be useful to extend subsequent studies to other clinical centres for the purpose of gaining a better insight into the specificity of each individual PICU stressor in different geographical areas. Secondly, the results of this study are based on quantitative collected data using a standardized questionnaire and using quantitative correlation methods, which can limit a complete deeper insight into certain aspects of parental stress in the PICU, especially from the aspect of causality.

### 4.2. Implications for Theory and Practice

This study contributes to the improvement of global knowledge about specific PICU stressors and a better understanding of the impact of certain stressors on parents during their stay in the PICU. Also, the results of this study may help nurses and other health professionals in assessing the stress level of parents in the PICU, but also in planning concrete strategies and methods for the purpose of preventing and minimizing their stressful experiences. Journal writing and the use of the PICU diary are among most economical, simple, feasible, and effective interventions in reducing psychological stress of parents and the entire family during the hospitalization of the child and alleviation of post-traumatic stress after discharge, since they enable parents to express feelings and concerns and help them to cope with traumatic events [49,50]. Journal writing is very popular among parents, and they themselves suggest such an approach to reducing stress to all parents whose child is hospitalized in the PICU [49]. A holistic and individualized approach must be part of daily nursing practice and family-centred care in the PICU. Finally, the results of this study should be useful academically as an incentive to other scientists, researchers, and clinicians to implement future similar studies in order to establish a more effective global comparability of the phenomenon of parental stress in the PICU.

### 4.3. Further Research and Practice

Further research should focus on specific PICU stressors in addition to the implementation of different research methods (quantitative, qualitative, and mixed) in order to ensure deeper insight into possible additional (hidden) stressors or possible cause-and-effect aspects of stress experienced by parents in the PICU. Also, future studies should focus on the development of regression models since they make it possible to determine total and individual contributions of individual and situational predictors of parental stress in the PICU (e.g., the child’s age, the child’s diagnosis, the prognosis of the child’s illness, the mental and physical health of the parents, the level of empathy and emotional competence of the parents, etc.), which will ensure studies’ predictive validity. Finally, it would be useful to compare the results of this and similar studies with the results of subsequent related studies that should focus on the improvement of parents’ mental health after the child’s hospitalization and their stay in the PICU.

## 5. Conclusions

Our study confirms the stressful experiences of the parents whose children were hospitalized in the PICU. The parents were exposed to numerous PICU stressors, and the highest level of experienced stress was expressed in the subscale ‘Parental role alteration’, while the lowest level of expressed stress was in the ‘Behaviours of professional staff’ subscale. The results of this study clearly point out the fact that the PICU is extremely stressful for parents and the whole family. Therefore, there is a global need to design measures and strategies for neutralization of individual PICU stressors, prevention of parental exposure to specific stressors, mitigating stressful experiences of parents in the PICU, providing professional psychological assistance to parents and the whole family, and finally establishing a system that will enable continuous assessment of parents’ stress level and timely prevention and minimization of stressful experiences for the parents in the PICU.

## Figures and Tables

**Table 1:** Sociodemographic characteristics of parents (N = 96).

Parental Characteristics		Number (%)
Gender	male	36 (37.5)
	female	60 (62.5)
Age (years)	20–25	5 (5.2)
	26–30	27 (28.1)
	31–35	28 (29.2)
	36–40	15 (15.6)
	41–45	15 (15.6)
	46–50	6 (6.3)
Place of Residence	urban	42 (43.52)
	rural	54 (56.3)
Level of Education	elementary school	6 (6.3)
	high school	60 (62.4)
	higher education	30 (31.3)
Profession	healthcare profession	27 (5.2)
	non-healthcare profession	91 (94.8)
Marital status	married	72 (75)
	divorced	18 (18.8)
	single	6 (6.3)
Number of Children	1	28 (29.2)
	2	39 (40.6)
	3	21 (21.9)
	4	5 (5.2)
	5	1 (1.0)
	6	2 (2.1)
Parents′ Experiences with Child′s PICU Hospitalization	first experience	62 (64.6)
	repeated experience	34 (35.4)
Self-assessment of Social Status	good	31 (32.3)
	satisfactory	57 (59.4)
	poor	8 (8.3)

**Table 2:** Level of perceived stress of parents according to the PSS: PICU subscales.

PSS: PICU Subscales	Mean	SD	*p* Value *
1. Child’s appearance	2.46	1.36	<0.01
2. Sights and sounds	2.82	1.39	
3. Procedures in PICU	2.69	1.38	
4. Staff communication	1.88	0.91	
5. Child’s behaviour and emotional responses	2.05	1.12	
6. Behaviours of professional staff	1.75	1.00	
7. Parental role alteration	3.00	1.30	
PSS: PICU In total	2.40	1.32	

* One-way analysis of variance.

**Table 3:** Exposure and level of perceived stress of parents according to PSS: PICU items.

PSS: PICU Subscales/PICU Stressors	Mean	SD	Rank	Number (%)
Subscale 1: Child’s appearance				
1. Puffiness of my child	2.68	1.14	16	70 (72.9)
2. Colour changes in my child (pale, blue, or yellow)	2.68	1.32	15	88 (91.6)
3. Child appearing cold	1.92	1.14	25	63 (65.6)
Subscale 2: Sight and sounds				
4. Seeing the heart beat on the monitors	2.74	1.32	12	95 (98.9)
5. The sound of monitors and equipment	2.59	1.33	19	93 (96.8)
6. The other sick children in the room	2.68	1.41	14	96 (100)
7. The sudden sounds of monitor alarms	3.24	1.43	3	95 (98.9)
Subscale 3: Procedures in PICU				
8. Injections/shots	2.38	1.13	20	93 (96.8)
9. Tubes in my child	2.90	1.42	9	93 (96.8)
10. Suctioning	2.59	1.62	18	74 (77.1)
11. Putting needles in my child for fluids, procedures, or tests	3.08	1.36	8	96 (100)
12. Making my child cough and deep breath and clapping on my child’s chest	1.89	0.99	27	63 (65.6)
13. Having a machine (respirator) breathe for my child	4.22	1.17	1	18 (18.8)
14. Bruises, cuts, incisions on my child	2.69	1.24	13	90 (93.8)
Subscale 4: Professional staff communication				
15. Explaining things too fast	1.65	0.80	36	54 (56.3)
16. Using words I don’t understand	2.04	0.80	24	78 (81.3)
17. Telling me different (conflicting) things about my child’s condition	2.05	0.96	23	75 (78.1)
18. Not telling me what is definitely wrong with my child	1.79	0.94	31	62 (64.6)
19. Insufficient communication with me	1.77	0.87	32	57 (59.4)
Subscale 5: Child’s behaviour and emotional responses				
20. Confusion	1.85	1.09	29	62 (64.6)
21. Rebellious or uncooperative behaviour	1.21	0.40	38	57 (59.4)
22. Crying or whining	2.81	1.37	11	96 (100)
23. Demanding	1.17	0.33	39	57 (59.4)
24. Acting or looking as if in pain	3.18	1.27	4	84 (87.5)
25. Restlessness	1.68	0.85	35	51 (53.1)
26. Inability to talk or cry	2.11	0.99	22	73 (76.0)
27. Fright	1.89	1.12	26	60 (62.5)
28. Anger	1.75	0.81	34	53 (55.2)
29. Sadness or depression	2.37	1.11	21	78 (81.3)
Subscale 6: Behaviour of professional staff				
30. Joking, laughing, or talking loudly	1.75	0.80	33	59 (61.5)
31. Not talking to me enough	1.79	0.99	30	56 (58.3)
32. Too many different people (doctors, nurses, staff) talking to me	1.85	1.02	28	68 (70.9)
33. Not telling me their names or who they are	1.54	0.91	37	46 (47.9)
Subscale 7: Parental role alteration				
34. Not taking care of my child myself	2.63	1.33	17	82 (85.4)
35. Not being able to visit my child when I wanted	3.09	1.27	7	88 (91.7)
36. Not being able to see my child when I wanted	3.14	1.25	5	88 (91.7)
37. Not being able to be with my crying child	2.86	1.40	10	81 (84.4)
38. Not being able to hold my child	3.10	1.26	6	88 (91.7)
39. Not knowing how best to help my child during this crisis	3.24	1.21	2	93 (96.9)

**Table 4:** The correlation between experienced stress and sociodemographic characteristics of the parents.

Sociodemographic Characteristics of Parents	Pearson Correlation Coefficient	*p* Value *
Age	0.118	0.251
Gender	−0.109	0.289
Place of residence	0.179	0.081
Marital status	−0.024	0.815
Number of children	0.240	0.019 *
Self-assessment of social status	−0.191	0.379

* p < 0.05.

**Table 5:** The level of perceived stress of parents with regard to their individual characteristics and previous experiences of child’s PICU hospitalization.

Individual Characteristics of Parents		PSS: PICU Subscales							PSS: PICUIn Total
		Child’s Appearance	Sights and Sounds	Proceduresin PICU	Staff Communication	Child’s Behaviour and Emotional Responses	Behaviours of Professional Staff	Parental role Alteration	
		Mean (SD)							
Level of Education	Elementary school	3.39 ± 0.90	4.08 ± 0.91	3.10 ± 0.67	1.73 ± 0.99	2.11 ± 1.12	1.38 ± 1.23	2.50 ± 1.13	2.67 ± 0.61
	High school	1.94 ± 1.32	2.78 ± 1.17	2.16 ± 1.11	1.36 ± 0.97	1.93 ± 0.98	1.10 ± 0.95	2.77 ±1.27	2.04 ± 0.90
	Higher education	1.46 ± 0.98	2.36 ±1.17	1.81± 0.96	1.03± 0.76	1.88 ± 0.78	0.83 ± 0.78	2.58 ±1.31	1.70 ± 0.80
	*p* value *	<0.01 ^†^	<0.01 ^†^	0.023 ^†^	0.133	0.302	0.281	0.739	0.032 ^†^
Profession	Healthcare	0.87 ± 0.38	1.75 ± 1.92	0.94 ± 0.51	0.12 ± 0.11	0.91 ± 0.22	0.05 ± 0.11	1.80 ± 0.97	0.93 ± 0.40
	Non-healthcare	1.94 ± 1.28	2.78 ± 1.16	2.17 ± 1.07	1.34 ± 0.92	2.28 ± 1.01	1.09 ± 0.92	2.75 ± 1.27	2.03 ± 0.87
	*p* value **	0.068	0.064	0.013 ^†^	<0.01 ^†^	0.024 ^†^	0.014 ^†^	0.106	<0.01 ^†^
Parents’ Experiences with Child’s PICU Hospitalization	First experience	1.65 ± 1.21	2.67 ± 1.13	1.90 ± 0.98	1.21 ± 0.79	1.61 ± 1.13	0.93 ± 0.86	2.49 ± 1.23	1.82 ± 0.82
	Repeated experience	2.31 ± 1.29	2.85 ± 1.37	2.48 ± 1.16	1.41 ± 1.15	2.24 ± 1.19	1.24 ± 1.01	3.08 ± 1.26	2.25 ± 0.93
	*p* value **	0.013 ^†^	0.491	0.011 ^†^	0.307	0.017 ^†^	0.120	0.028 ^†^	0.022 ^†^

* One-way analysis of variance; ** Unpaired Student’s *t*-test; ^†^ *p* < 0.05.

## Data Availability

All data generated analyzed during the current study are available from the corresponding author on reasonable request.
